# Virtual Screening for HIV Protease Inhibitors: A Comparison of AutoDock 4 and Vina

**DOI:** 10.1371/journal.pone.0011955

**Published:** 2010-08-04

**Authors:** Max W. Chang, Christian Ayeni, Sebastian Breuer, Bruce E. Torbett

**Affiliations:** 1 Department of Molecular and Experimental Medicine, The Scripps Research Institute, La Jolla, California, United States of America; 2 Department of Bioengineering, University of California Merced, Merced, California, United States of America; University of Cambridge, United Kingdom

## Abstract

**Background:**

The AutoDock family of software has been widely used in protein-ligand docking research. This study compares AutoDock 4 and AutoDock Vina in the context of virtual screening by using these programs to select compounds active against HIV protease.

**Methodology/Principal Findings:**

Both programs were used to rank the members of two chemical libraries, each containing experimentally verified binders to HIV protease. In the case of the NCI Diversity Set II, both AutoDock 4 and Vina were able to select active compounds significantly better than random (AUC = 0.69 and 0.68, respectively; p<0.001). The binding energy predictions were highly correlated in this case, with r = 0.63 and ι = 0.82. For a set of larger, more flexible compounds from the Directory of Universal Decoys, the binding energy predictions were not correlated, and only Vina was able to rank compounds significantly better than random.

**Conclusions/Significance:**

In ranking smaller molecules with few rotatable bonds, AutoDock 4 and Vina were equally capable, though both exhibited a size-related bias in scoring. However, as Vina executes more quickly and is able to more accurately rank larger molecules, researchers should look to it first when undertaking a virtual screen.

## Introduction

The use of virtual screening to discover new inhibitors is becoming a common practice in modern drug discovery [1]. Receptor-based virtual screens seek to “dock” members of a chemical library against a given protein structure, predicting the conformation and binding affinity of the small molecules [2]. A large number of programs are available for this purpose, such as DOCK [3], FlexX [4], GOLD [5], and AutoDock [6], [7], [8]. This study focuses on AutoDock 4 and AutoDock Vina (henceforth referred to as AD4 and Vina), both notable for being among the few docking programs that are freely available for academic and industrial use. The AutoDock programs are further unique in that they are some of the only widely-used docking programs released under open source licenses (GNU General Public License and Apache Open Source License).

**Figure 1:**
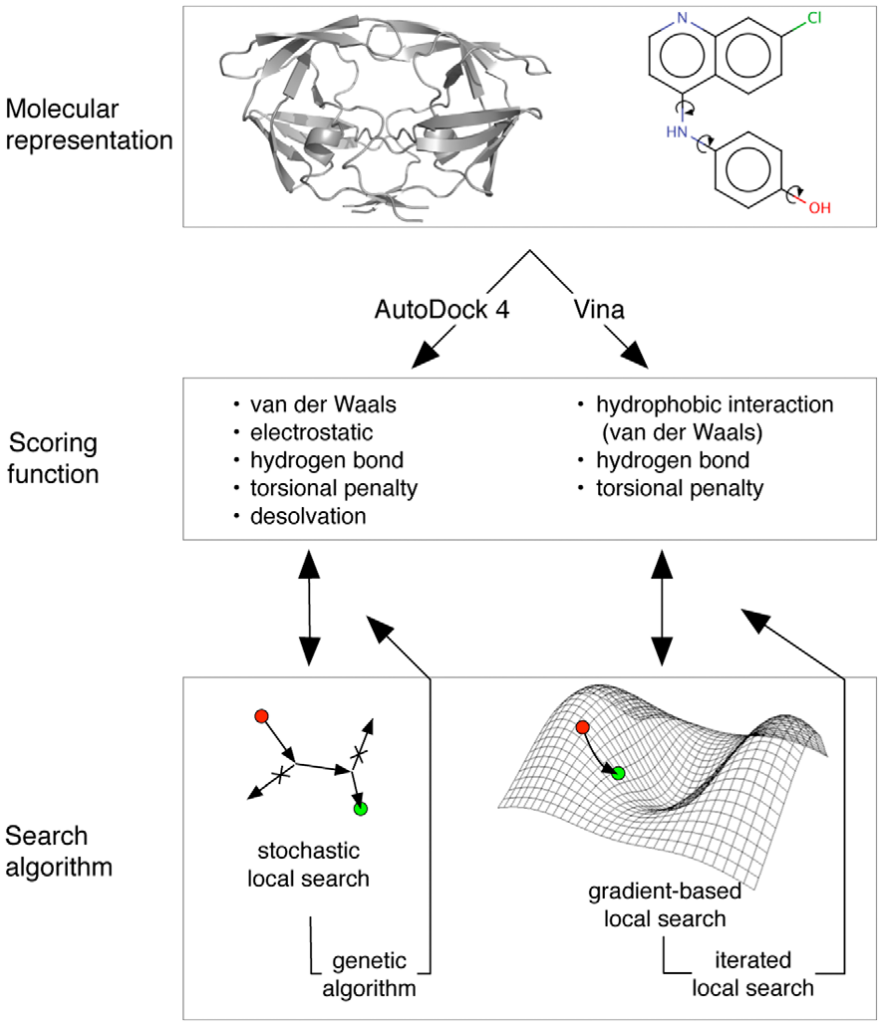
Comparing the methodologies of AD4 and Vina. Both programs use the same type of input files describing the receptor (generally rigid) and flexible ligand. The scoring functions have similar parameters, but have been calibrated differently. A key difference between the programs is the local search algorithm. AD4 uses a stochastic search which generates random conformations to test. Vina calculates a gradient while seeking a local optimum.

Both AD4 and Vina operate in a roughly similar manner, pairing an empirically-weighted scoring function with a global optimization algorithm. Key differences lie in the local search function (illustrated in Figure 1) and parameterization of the scoring function. In addition, Vina is designed to operate much more quickly and its authors have shown that its accuracy in re-docking protein-ligand complexes is greater than AD4 [8]. For 190 protein-ligand complexes, Vina was able to recapitulate the observed binding mode within 2 Å RMSD in 78% of cases, while AD4 succeeded for only 49%. However, using AD4 and Vina to screen chemical libraries was not addressed. In this study, we compared the ability of AD4 and Vina to identify ligands by ranking the relative binding affinity of small molecules.

For this task, the National Cancer Institute (NCI) Diversity Set II (DSII) was one of the chemical libraries used. DSII contains 1,364 compounds that tend to be small (the average molecular weight is less than 300 Daltons) and have few rotatable bonds. HIV protease was chosen as the protein target because it is a well-studied protein that has been a major focus for structure-based drug design [9]. As a complement to the relatively small DSII compounds, an additional collection of molecules was taken from the Directory of Universal Decoys (DUD) [10]. DUD contains known ligands for a variety of proteins, and provides accompanying “decoys” – molecules with composition similar to the known ligands, but with a different topology – that are assumed not to bind to the protein. There are 53 known HIV protease ligands in DUD, along with 1,885 decoys. Overall, these compounds tend to be appreciably larger than those from DSII, in terms of both molecular weight and number of rotatable bonds.

Although DUD is already divided into known “active” and inactive compounds against HIV protease, that information is not available for DSII. A biophysical method, differential scanning fluorimetry (DSF) [11], [12], [13], was used to infer binding between HIV protease and the constituents of DSII. DSF functions by measuring the melting temperature of a protein through the use of a fluorescent dye that interacts with the hydrophobic regions of the protein. As a protein in solution is heated in the presence of this dye, the protein unfolds and more of its surface is exposed to the dye, which generates a greater fluorescent signal. The melting temperature can be determined based on fluorescence measurements taken during a gradual increase in temperature. The presence of a bound ligand will stabilize the protein, increasing the melting temperature. Screening DSII via DSF revealed a number of stabilizing ligands, which were in turn treated as active compounds for the virtual screen. The DSF assay does not provide information on the binding site of the ligand, so the docking studies focused on the selection of active compounds rather than specific binding modes.

To evaluate the performance of AD4 and Vina in ranking the small molecules from DSII and DUD, each compound was docked against a single HIV protease structure. The predicted binding energy from the dockings provided a ranking of the compounds, which was compared to the known actives using two measures. Virtual screening performance is commonly analyzed using a receiver operating characteristic (ROC) curve, which can easily be quantified by determining the area under the curve (AUC). The AUC, as well as the Boltzmann-enhanced discrimination of receiver operating characteristic (BEDROC) metric, were used to evaluate the ability of the docking programs to select active compounds [14]. In the following sections, we examine the results from docking the DSII and DUD libraries to contrast the performance of AD4 and Vina, analyze similarities and differences in their predictions, and offer recommendations for users of these programs.

## Results

### DSF screen for HIV protease ligands

In an effort to identify new inhibitors of HIV protease, a biophysical method, DSF, was used to identify compounds which altered the melting temperature of a protease sample. Such an observation suggests that a compound binds protease, though it may not act as an inhibitor. Additionally, no information regarding a binding site is provided. In a first pass, all 1,364 compounds of DSII were assayed individually. The 84 compounds which initially indicated a thermal shift were subsequently re-screened in triplicate. Of these, 25 compounds (shown in Table S1) displayed consistent shifts of at least 0.6°C beyond the control. These 25 compounds comprised the active set used to evaluate the virtual screen in the following section.

### Virtual screen of NCI Diversity Set II

Using AD4 and Vina, the 1,364 members of DSII were docked against HIV protease. From the results of each program, the compounds were ranked based on their predicted binding energies. These rankings were used to evaluate the ability of AD4 and Vina to preferentially select the active compounds as classified by DSF. Based on a previous study, the 2BPW structure was found to be representative of wild-type HIV protease and was used as the receptor in our investigations [15]. A large bounding box was used, which encompassed the entire protein. In general, the default parameters were used for both AD4 and Vina. Each docking program reported multiple conformations and associated binding energies. In the case of AD4, the results were processed by the built-in clustering analysis, and the lowest energy conformation from the largest cluster chosen as representative. For Vina, the lowest energy conformation was selected. The compound rankings were determined for each program, then compared against the 25 compounds designated as active by the DSF screen.

**Figure 2:**
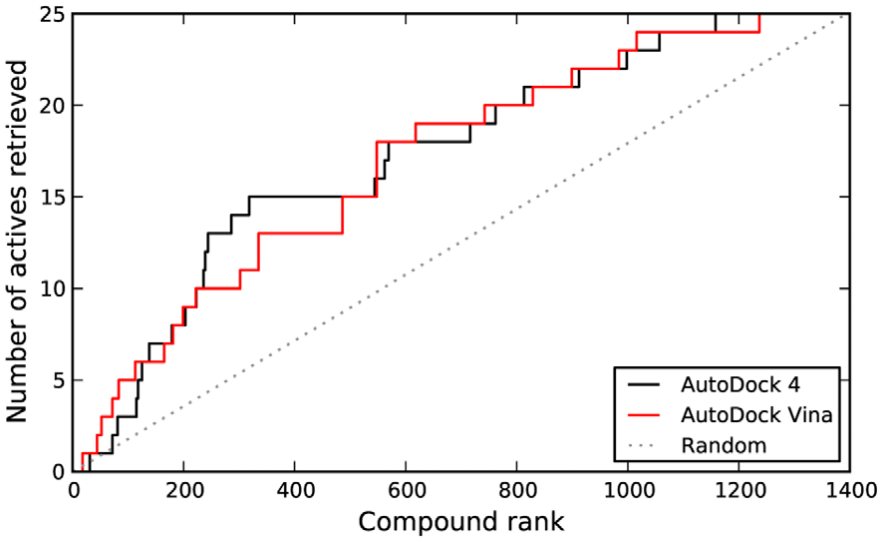
Virtual screen ranking of NCI Diversity Set II. The 1,364 members of DSII were docked to HIV protease using AD4 and Vina, then ranked by predicted binding energy. The plot shows the number of active compounds (determined via DSF) retrieved versus the total number selected. The dashed line indicates the number of actives that would be expected to be returned based on a random selection of compounds.

As shown in Figure 2, AD4 and Vina displayed similar performance in correctly ranking active compounds in DSII. Quantified by an AUC measure (Table 1), AD4 had a slight edge over Vina, but both were highly significant when compared to random rankings. In terms of early recognition, determined using the BEDROC measure, only Vina seemed to perform significantly better than random.

**Table 1:** Virtual screen statistics.

Library		AUC	p-value	BEDROC 20	p-value
NCI Diversity Set II	AutoDock 4 AutoDock Vina	0.69 0.68	0.00043 0.00091	0.11 0.14	0.10 0.028
DUD HIV protease set	AutoDock 4 AutoDock Vina	0.40 0.66	— 0.00015	0.077 0.16	0.30 0.0014

Area under the curve (AUC) and Boltzmann-enhanced discrimination of receiver operating characteristic (BEDROC) 20 values were calculated based on the data shown in Figures 2 and 6. P-values were estimated using a bootstrap procedure based on 100,000 random rankings of the active compounds.

**Figure 3:**
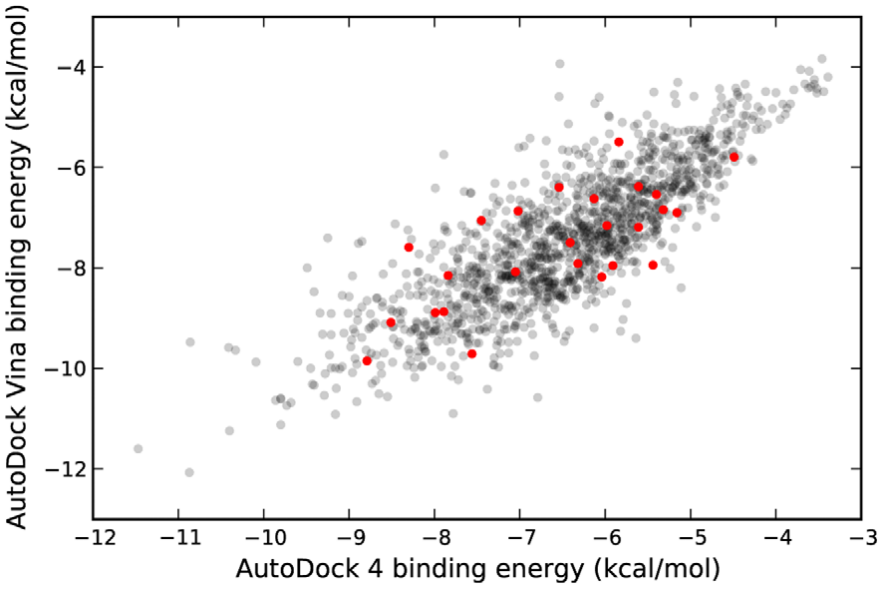
Predicted binding energies for NCI Diversity Set II compounds with HIV protease as determined by AD4 and Vina. A moderately strong correlation was observed (*r* = 0.63, *p*≪0.0001). A small amount of random noise (<0.1 kcal/mol) was added to Vina binding energies for visual effect. Active compounds are highlighted in red.

**Figure 4:**
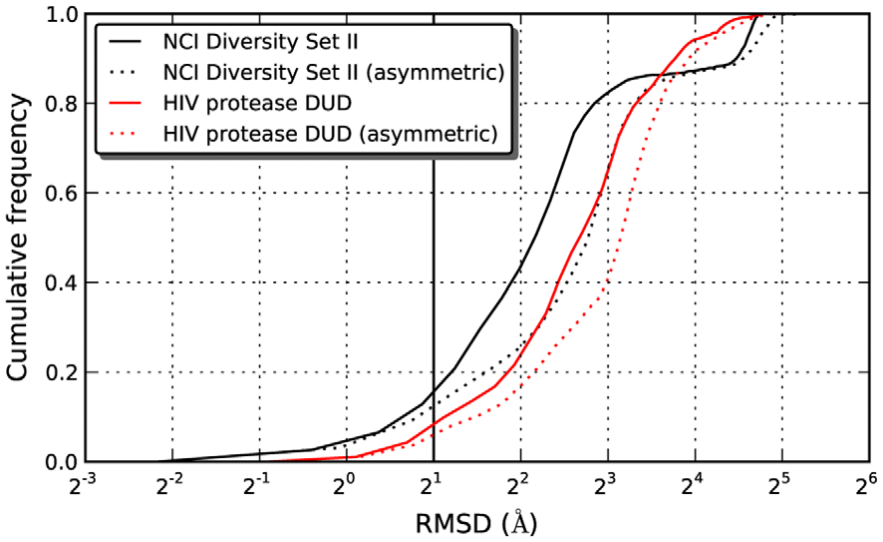
Cumulative RMSD distribution between AD4 and Vina conformations. Differences in the binding conformations predicted by AD4 and Vina were compared using RMSD. As HIV protease is a homodimer, calculations are shown with and without regard for protein's symmetry. Only a small fraction of compounds fall under the 2 Å threshold traditionally used to indicate close agreement in conformational space.

A comparison of the predicted binding energies from both programs is shown in Figure 3, demonstrating a marked correlation between the docking results. As evidenced by both Kendall rank correlation and traditional Pearson correlation (Table 2), there was a clear association between the predictions from AD4 and Vina. Based on this correlation in terms of binding energy, it was expected that the conformations reported by both programs would also tend to be similar. However, pairwise comparisons of the docked conformations reported by AD4 and Vina showed that most of the compounds differed by more than 4 Å RMSD (Figure 4). Because HIV protease consists of two identical subunits arranged in a symmetric manner, RMSD calculations may be exaggerated when the symmetry is not taken into account. In other words, a ligand conformation interacting with chain A should be considered identical to the equivalent conformation bound to chain B. Even allowing for symmetry, though, the conformations tended to be quite different.

**Table 2:** Correlation between AutoDock 4 and Vina.

	Correlation	p-value
Pearson (r)	0.63	≪0.0001
Kendall (τ)	0.82	≪0.0001

**Figure 5:**
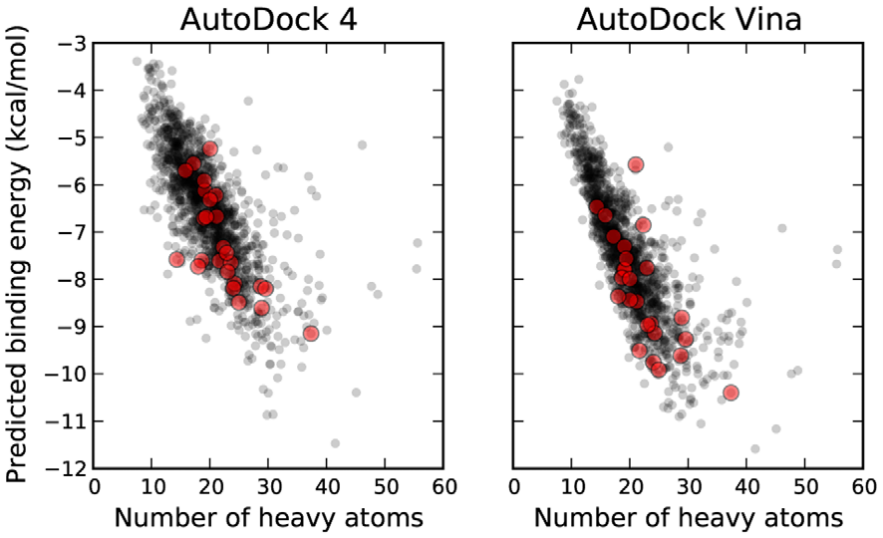
Predicted binding energies for NCI Diversity Set II compounds with HIV protease as a function of the number of heavy atoms in the compound. *r* = −0.75 and *r* = −0.79 for AD4 and Vina, respectively. Active compounds are highlighted in red.

Finding it curious that the results were similar in binding energy, but very dissimilar in terms of conformation, we turned to an analysis of the properties of the compounds. Historically, protein-ligand docking programs have been susceptible to bias based on the size of the compound [16]. A comparison of the number of heavy atoms present in each compound plotted against the predicted binding energy of each compound revealed strong correlations for both AD4 and Vina (Figure 5). For relatively small compounds, then, it appears that the binding energy predictions are strongly influenced by size alone, though both programs favored the active compounds to a significant extent.

### Virtual screen of DUD library

In contrast to DSII, the DUD compounds tended to be larger in size and, by design, more homogeneous. From a docking standpoint, these compounds also posed more of a challenge, as the average number of rotatable bonds was 9.7 for the DUD compounds, compared to 3.7 for DSII (Figure S1). The 53 active compounds and 1,885 decoys from DUD were docked to the 2BPW HIV protease structure and the results processed in the same manner as the DSII compounds detailed above.

**Figure 6:**
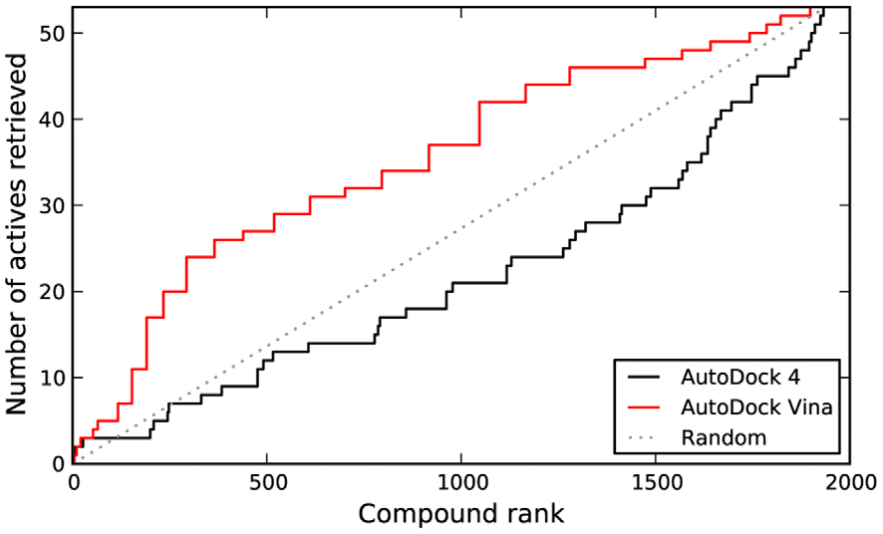
Virtual screen ranking of DUD compounds. The 1,885 DUD compounds were docked to HIV protease using AD4 and Vina, then ranked by predicted binding energy. The plot shows the number of active compounds (as designated by DUD) retrieved versus the total number selected.

**Figure 7:**
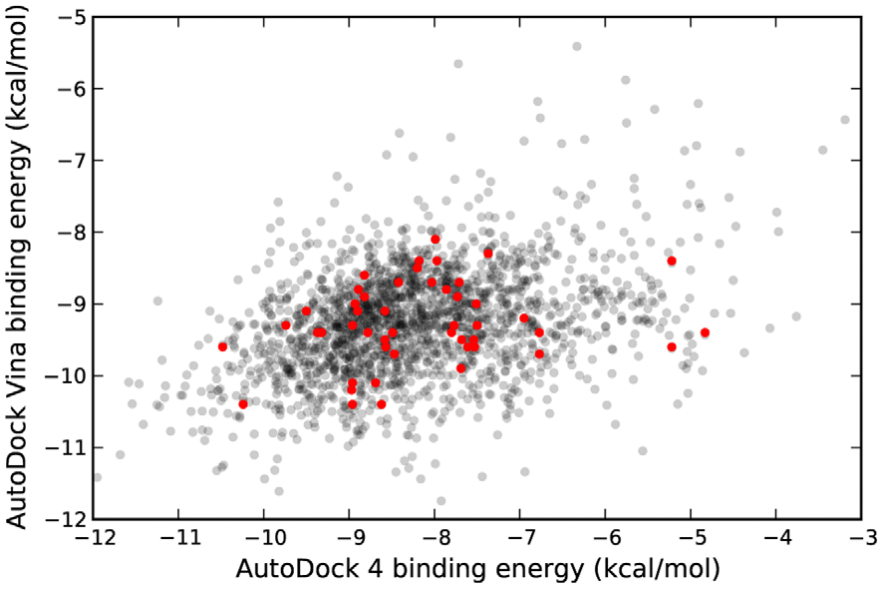
Predicted binding energies for DUD compounds with HIV protease as determined by AD4 and Vina. No significant correlation was observed. Active compounds are highlighted in red.

**Figure 8:**
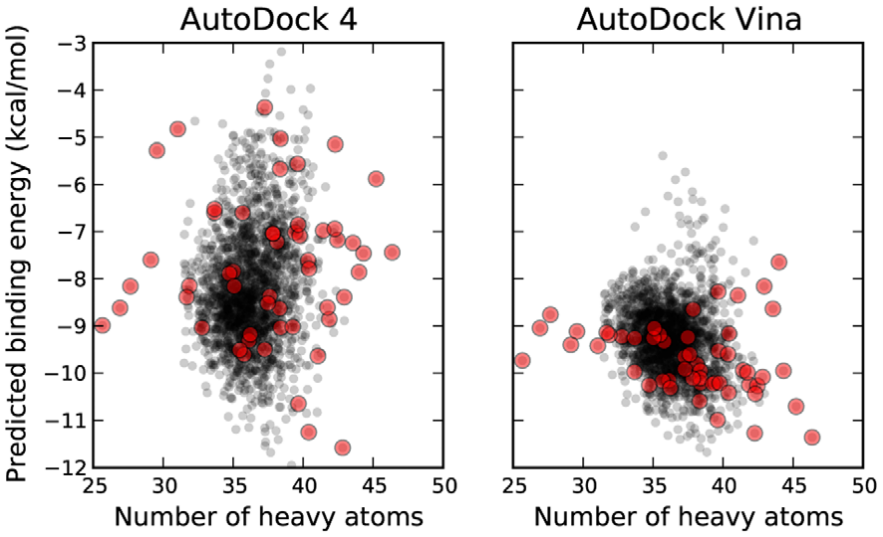
Predicted binding energies for DUD compounds with HIV protease as a function of the number of heavy atoms in the compound. Active compounds are highlighted in red.

Unlike what was seen with DSII, Vina showed clear superiority over AD4, which performed worse than random selection (Figure 6). Interestingly, both the AUC and BEDROC values for Vina's performance, shown in Table 1, were very similar to those obtained from the experiments with DSII. In this screen, no significant correlation between AD4 and Vina binding energies was found, as shown in Figure 7. Likewise, neither program displayed a strong correlation between the number of heavy atoms in the compounds and the predicted binding energies, as was seen with the DSII compounds (Figure 8).

In general, AD4 and Vina reported highly disparate conformations for the DUD compounds. This occurred to an even greater extent than was seen previously with DSII, as shown in Figure 3. Based on the larger size of the compounds and greater number of rotatable bonds in DUD, it seemed possible that AD4 would possibly fail to even find the most favorable conformations consistently. As each compound was docked in 100 independent trials with AD4, cluster analysis provided a way to analyze variations in the reported conformations. The distribution of cluster sizes (Figure S2) shows that the docked conformation from DSII tended to fall into large clusters, while those from DUD did not. Small clusters indicate that AD4 had difficulty in consistently determining binding modes for the larger compounds in the DUD library.

### Comparing AutoDock 4 and Vina Methodologies

To explore the differences between AD4 and Vina in docking the DUD library, we explored the methodology of each program in detail. In a broad sense, the advantage of Vina over AD4 in addressing larger molecules must be due to one or more of the major components of a docking program: 1) molecular representation, 2) scoring function, and 3) search algorithm. As AD4 and Vina both use the same input files for the receptor and ligand, differences in representation are not a factor. The scoring functions and search algorithms, on the other hand, share similarities in overall form, but have distinct implementations.

**Figure 9:**
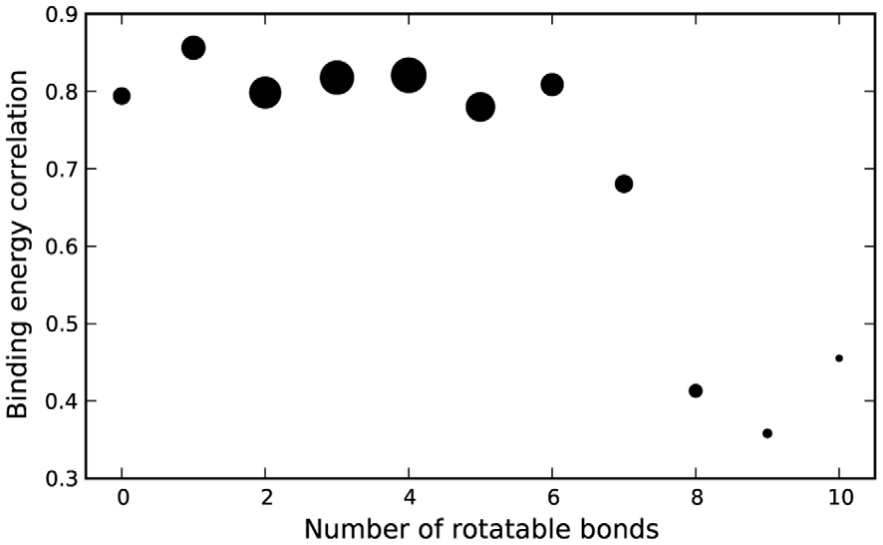
Correlation in scoring between AD4 and Vina. Identical conformations of DSII ligands were evaluated using AD4 and Vina. Correlations between these scores was organized by the number of rotatable bonds in the ligands, indicating increasingly dissimilar scores as the number of rotatable bonds grew. The area of each circle is proportional to the number of ligands that contained the specified amount of rotatable bonds.

The scoring functions, for instance, are both empirically weighted functions containing terms for values such as hydrogen bonding and rotatable bond penalties. While there are obvious differences in these parameters [7], [8], it was unclear if the overall scores would also differ. Due to differences in the programs' methodologies, there were limited possibilities to de-couple scoring and search, and so we focused on determining the degree of correlation between AD4 and Vina in scoring identical ligand conformations. Using AD4's ability to score arbitrary ligand conformations, we evaluated each of the final conformations reported by Vina. Ligands were grouped by their number of rotatable bonds, and the correlation between AD4 and Vina energies for all conformations within each group was calculated. Any positive energy values, which occurred with a frequency of less than 1%, were ignored. As shown in Figure 9, the correlation in scores for ligands with 6 or fewer rotatable bonds was generally greater than 0.8, while the correlation dropped below 0.5 for ligands with 8 or more rotatable bonds. Since the number of rotatable bonds is primarily associated with a larger search space, it was surprising to observe a difference in scoring as well.

In terms of the search algorithm, both programs apply a hybrid global-local search, but the key difference appears to be in the local optimization. The local search method in AD4 employs small random steps while seeking more favorable conformations. No gradients are calculated, though the size of the steps is adjusted. In contrast, Vina calculates derivatives to generate a gradient, performing its optimization accordingly. For technical reasons, evaluating the search algorithms of AD4 and Vina independently of their scoring functions was not feasible. However, the small clusters generated by AD4 dockings while evaluating the DUD library (Figure 8) show that AD4 had difficulty reliably finding consistent energy minima. Absent any consideration of the scoring function, this behavior indicates that the search algorithm is ineffective for molecules with a large number of rotatable bonds. Vina does not provide a cluster analysis, but its authors have demonstrated superior performance over AD4 in reproducing experimentally observed binding modes as the number of rotatable bonds grows [8].

## Discussion

The virtual screening abilities of AD4 and Vina were compared based on binding predictions between the members of two chemical libraries and HIV protease. DSII contained molecules that were generally small, with few rotatable bonds, and both programs were able to select active compounds with a similar, significant level of accuracy. A strong correlation was found between the results of the two programs, as well as between the predicted binding energies and the number of heavy atoms present in the compounds. Coupled with the low agreement in conformational similarity, however, it appears that similarity in the binding energy predictions from both programs suffer from a size-related bias in scoring, and that AD4 and Vina report distinct results.

A clear advantage for Vina was noted in the virtual screen of the DUD library, whose constituents tended to be larger molecules, with more rotatable bonds than DSII. As the search space in protein-ligand docking is related exponentially to the number of rotatable bonds present, this presented a far more difficult docking problem. AD4 failed to preferentially rank active compounds, while Vina maintained performance comparable to the results from the DSII screen. Accordingly, it seems that Vina is more scalable in addressing more difficult docking problems (i.e., larger, more flexible compounds) than AD4.

In comparison, other popular docking programs also have difficulty with increasingly flexible ligands. A 2004 study found that DOCK, FlexX, and GOLD could reproduce the binding modes of an assorted set of protein-ligand complexes with reasonable accuracy (> = 70%) when the ligands had fewer than 8 rotatable bonds [17]. However, for ligands with 8 or more rotatable bonds, none of the programs was able to reproduce observed binding modes with even 30% accuracy.

Based on the docking results as a whole, both AD4 and Vina are capable of providing useful predictions when modeling compounds with a small number of rotatable bonds. However, based on the results with larger compounds, users should look to Vina first when undertaking a virtual screen. Vina's other strengths include streamlined parameters and much faster docking performance. In this study, docking each library required approximately 10 times longer with AD4 compared to Vina.

Some users may still be well served by AD4, which benefits from a long heritage. For instance, the MGLTools suite contains a feature-rich GUI which can guide users through the process of setting up a docking run or analyzing docking results. The open source nature of AD4 has allowed customization for a wider variety of problems, such as RNA-ligand docking [18]. For users of AD4, a limit of 10 rotatable bonds may serve as a rough guide for protein-ligand docking problems, though this would depend on the size and properties of the binding site.

## Materials and Methods

### Differential scanning fluorimetry

The DSF compound screen generally followed the protocol published by Niesen et al. [11]. Samples were loaded into a white 96-well PCR plate (Bio-Rad) with each well containing a final volume of 40 µl. The concentration of HIV protease in each well was 1.25 µM (26.8 µg/ml), with 10 mM pH 7.5 HEPES, 150 mM NaCl, and 5X SYPRO Orange (Invitrogen). DSII compounds were used at a concentration of 250 µM, taken from stocks supplied by the NCI as 10 mM solutions dissolved in DMSO. The PCR plates were sealed with optical quality sealing tape (Bio-Rad).

DSF experiments were carried out using an iCycler iQ real-time PCR system (Bio-Rad) set to use the 490/20 excitation and 575/20 emission filters. The samples were heated from 20 to 95°C at the rate of 1°C/minute. A single fluorescence measurement was taken each minute. Melting temperatures were determined by performing a curve fit to the Boltzmann equation. The degree of thermal shift was calculated by comparing the melting temperature of the protease in the presence of various DSII compounds against a negative control that contained DMSO.

### Ligand and receptor preparation

Structural representations of the NCI Diversity Set II in SMILES format were obtained from the Developmental Therapeutics Program website. CORINA [19], via the NCI's Online SMILES Translator and Structure File Generator, was used to generate 3-dimensional coordinates in PDB format from the SMILES-formatted file. 36 of the compounds contained arsenic atoms, which are not supported by AD4 or Vina, so all arsenic atoms were replaced by phosphorus. Individual PDB files were prepared for docking using the prepare_ligand4.py script from MGLTools 1.5.4 [20], using only the largest non-bonded fragment present.

The DUD decoys and ligands for HIV protease were obtained from the DUD website. These mol2-formatted files already contained 3-dimesional coordinates, and were translated to PDB format using Open Babel 2.2.3 [21]. Following conversion to PDB format, files containing individual compounds were created, then processed using MGLTools.

The 2BPW HIV protease structure was obtained from the PDB [22]. To prepare the structure for docking, the ligand and all water molecules were removed. Charges and non-polar hydrogen atoms were added using the prepare_receptor4.py script from MGLTools.

### Docking parameters

AutoDock 4.2.1 and Vina 1.0.2 were used for all dockings in this study. In general, the docking parameters for both AD4 and Vina were kept to their default values. However, the number of AutoDock 4 GA runs was increased from 10 to 100 and the grid spacing changed from 0.375 to 0.5. For both AD4 and Vina, the size of the docking grid was 63 Å×47 Å×40 Å, which encompassed the entire HIV protease structure. The 100 independent GA runs from AD4 were processed using the built-in clustering analysis with a 2.0 Å cutoff.

### Analysis of docking results

Virtual screen performance was quantified using AUC and BEDROC measures. AUC was calculated via summation, while BEDROC 20 values were obtained using the Python code provided by Truchon and Bayly [14]. To estimate the statistical significance of these results, a bootstrap method was applied. Random rankings for *m* compounds were sampled from the range 1…*n*, where *m* is the number of active compounds and *n* is the number of compounds in the library. These random rankings were used to calculate AUC and BEDROC values, and the process repeated 100,000 times.

## Supporting Information

Table S1 DSF hits. NSC ID numbers for compounds designated as active in DSF screen.
(0.02 MB XLS)

Figure S1 Distribution of rotatable bonds in NCI Diversity Set II and DUD compounds.
(0.03 MB EPS)

Figure S2 Distribution of sizes for largest clusters in AD4 results. Each compound was docked in 100 independent AD4 runs, then clustered based on conformation, serving as an indication of the level of convergence of the docking results.
(0.02 MB EPS)
